# Simvastatin Ameliorates Liver Fibrosis via Mediating Nitric Oxide Synthase in Rats with Non-Alcoholic Steatohepatitis-Related Liver Fibrosis

**DOI:** 10.1371/journal.pone.0076538

**Published:** 2013-10-02

**Authors:** Wei Wang, Caiyan Zhao, Junying Zhou, Zhen Zhen, Yadong Wang, Chuan Shen

**Affiliations:** Department of Infectious Diseases, the Third Affiliated Hospital of Hebei Medical University, Shijiazhuang, Hebei Province, China; CIMA. University of Navarra, Spain

## Abstract

**Background:**

Simvastatin exerts pleiotropic effects on cardiovascular system. However, its effect on non-alcoholic fatty liver disease, especially the liver fibrosis, remains obscure. We aimed to clarify the relationship between simvastatin and liver fibrosis both in vivo and in vitro.

**Methods:**

A High-fat diet was given to establish rat models with non-alcoholic steatohepatitis (NASH)-related liver fibrosis and simvastatin (4mg·kg^-1^·d^-1^) was administrated intragastrically until hepatic histological findings confirmed the appearance of fibrosis. Human hepatic stellate cell (HSC) line lx-2 cells were cultured in an adipogenic differentiating mixture (ADM) and then were treated with transforming growth factorβ1 (TGF-β1), served as a positive control, simvastatin, TGF-β1 plus simvastatin, N_ω_-nitro-L-arginine methyl ester hydrochloride (L-NAME, a inhibitor of nitric oxide synthase), and L-NAME plus simvastatin, respectively. The expressions of endothelial nitric oxide synthase (eNOS), inducible nitric oxide synthase (iNOS), and Collagen І as well as cellular α-smooth muscle actin (α-SMA) were measured by real-time reverse transcriptase-polymerase chain reaction (qRT-PCR) and Western blot in liver tissue and HSC.

**Results:**

With the progress of NASH-related fibrosis, hepatic mRNA and protein expressions of iNOS, α-SMA, and Collagen І were increased while those of eNOS were decreased. Compared with model rats in 24^th^ week group, rats in simvastatin group had less expressions of iNOS, α-SMA, and Collagen І and more expressions of eNOS. In vitro, LX-2 cells acquired quiescent phenotype when cultured in ADM, and TGF-β1 could activate the quiescent HSC. Simvastatin inhibited LX-2 cells activation due to TGF-β1 or L-NAME by increasing the expression of eNOS and decreasing the expression of iNOS.

**Conclusions:**

Simvastatin improves the prognosis of NASH-related fibrosis by increasing the expression of eNOS, decreasing the expression of iNOS, and inhibiting the activation of HSC.

## Introduction

Non-alcoholic fatty liver disease (NAFLD) has become a growing public health concern and been considered to be the most common cause of chronic liver disease in Western countries [1-4]. In China, it is increasingly diagnosed as well. NAFLD involves a histopathological spectrum ranging from benign simple steatosis to non-alcoholic steatohepatitis (NASH), fibrosis, cirrhosis, and even malignant hepatocellular carcinoma [5-11]. The profibrogenic mechanisms operating in NASH are complicated, and insulin resistance, oxidative stress, altered cytokines, especially adipokines, might play important roles in the fibrogenesis in NASH-related fibrosis [7,8]. Many studies have reported that transforming growth factor β1 (TGF-β1) is increased in serum and hepatic tissues in the NAFLD patients and animal models [12,13]. It can promote hepatic fibrogenesis by activating hepatic stellate cells (HSC) in both autocrine and paracrine way. HSC is recognized as the major source of extracellular matrix (ECM), of which increased or altered deposition can lead to fibrosis and severe cirrhosis.

Recently, statins have been considered to exert pleiotropic effects on cardiovascular system [14]. Researchers found that statins, such as pitavastatin, atorvastatin, and rosuvastatin, could improve the activity of NAFLD by ameliorating the hepatic steatosis, hepatitis, and fibrosis [15-17]. Simvastatin was also reported to lower the elevated liver enzymes and reduce hepatic fatty infiltration in patients with NAFLD [18], and to stabilize or reverse fibrosis [19] by inhibiting HSC proliferation [20]. However, many other studies obtained the negative outcomes or the converse results. Therefore, it is still controversial that whether simvastatin has the therapeutic effect on NAFLD, particularly on NASH-related hepatic fibrosis.

Nitric oxide (NO) is generated constitutively from sinusoidal endothelial cells mediated by endothelial NO synthase (eNOS) under normal physiological conditions [21]. eNOS-derived NO exerts paracrine effects on adjacent HSC, inhibiting the vasoconstriction, proliferation, and migration. During the inflammation, inducible NO synthase (iNOS) plays a major role in NO production contributing to tissue damage [22]. Simvastatin has been concerned to increase eNOS activity, enhance NO bioavailability, and prevent a significant increase in iNOS in rats after ischemia-reperfusion [23]. In addition, HSC has the ability to excrete a little content of eNOS in normal condition. Therefore, we presume that simvastatin might inhibit the activation of HSC by increasing eNOS expression and decreasing iNOS expression.

In this study, we aim to demonstrate whether simvastatin exert an antifibrogenic effect on rats with NASH-related hepatic fibrosis and how it works.

## Materials and Methods

### Reagents

3-Isobutyl-1-methylxanthine, dexamethasone, insulin, Dulbecco’s modified Eagle’s medium (DMEM), N_ω_-Nitro-L-arginine methyl ester hydrochloride (L-NAME), simvastatin were purchased from Sigma-Aldrich (Saint Louis, MO, USA), fetal bovine serum (FBS) was purchased from Gibco (Langley, OK, USA), recombinant human transforming growth factor β1 (TGF-β1) was obtained from Peprotech (Rocky hill, NJ, USA), Trizol reagent was obtained from Invitrogen (Carlsbad, CA, USA), reverse transcription system and oligonucleotide primers were obtained from Promega (Madison, WI, USA), iTaq SYBR Green supermix used for PCR were purchased from Bio-Rad (Hercules, CA, USA), antibodies against eNOS, iNOS, α-smooth muscle actin (α-SMA), Collagen І, and β-actin, and secondary antibodies were all purchased from Santa Cruz (Santa Cruz, CA, USA).

### Rat models with NASH-related hepatic fibrosis

Forty-eight male Wistar rats weighting from 140 grams to 160 grams were approved from the experimental animal center of Hebei medical university. The animals were maintained on a controlled temperature (20°C-24°C) and humidity (65%-75%), and they had free access to food and water. After one week of acclimatization, all the 48 rats were divided randomly into two groups, the control group fed with standard diet and the model group fed with high-fat diet (standard diet plus 10% lard, 2% cholesterol, and 5% corn oil). There were 12 rats in the control group and 36 in the model group. Of 36 rats with high-fat diet, six rats were respectively sacrificed at the ends of 8^th^, 12^th^, 16^th^ week by bloodletting at femoral vein. Hepatic histological examinations confirmed that liver fibrosis appeared at the end of 16^th^ week. Since then, six rats were separated randomly from the rest of 18 rats in the model group and started to treat with simvastatin intragastrically besides the high-fat diet. At the ends of 20^th^ week, six rats were sacrificed randomly in the model group. And at the ends of 24^th^ week, the rest six rats with high-fat diet and six rats with simvastatin plus high-fat diet were all sacrificed. Of 12 rats in the control group, two were sacrificed respectively at the end of 8^th^, 12^th^, 16^th^, 20^th^ week, and four were sacrificed at the end of 24^th^ week. The serums were collected to examine the serum biochemical markers, and liver specimens were obtained to observe hepatic pathological changes and to detect the hepatic mRNA and proteins.

All animal protocols and practices were reviewed and approved by the Hebei Medical University Experimental Animal Center. All rats were anesthetized with 1% sodium pentobarbital given i.p. before sacrificed by bloodletting at femoral vein, and they received humane cares indeed which minimize animal pain or discomfort.

### Cell culture

Human HSC line LX-2 cells from the cell bank of Chinese Academy of Sciences were cultured in DMEM containing 10% FBS, penicillin (100 U/ml), and streptomycin (100 µg/ml) at 37°C with 5% CO_2_. Cells were seeded in six well plates at a density of 5 × 10^5^ cells per well until 70% cells confluent, and then treated with the adipogenic differentiation mixture (ADM), which containing 0.5 mM 3-Isobutyl-1-methylxanthine, 1 µM dexamethazone, and 1 µM insulin. Seventy-two hours later, ADM was changed to DMEM with 0.2% FBS, penicillin, and streptomycin for 24 hours to achieve cell synchronization. After that, cells were respectively treated with solvent (the control group), TGF-β1 (100 pM, the positive control group), simvastatin (10 µM), TGF-β1 plus simvastatin, L-NAME (100 µM), and L-NAME plus simvastatin for 24 hours, and were harvested to assess the expressions of eNOS, iNOS, α-SMA, and Collagen I.

### Biochemical assay

Serum alanine aminotransferase (ALT), aspartate aminotransferase (AST), cholesterol (TC), and triglyceride (TG) of rats were measured using an auto-biochemical analyzer OLYMPUS AU-600 from Olympus Corporation (Shinjuku, Japan).

### Staining

Some of liver specimens were made of frozen sections and were stained by Sudan IV for hepatic steatosis. Another part was fixed with 10% formalin and then was made of paraffin sections. Hematoxylin-eosin (HE) staining was used for observing the general hepatic pathological changes, particularly hepatocellular ballooning degeneration, inflammatory cells infiltration, and necrosis, and Masson staining for fibrosis.

### Quantitative real-time reverse transcriptase-polymerase chain reaction (qRT-PCR) analysis

Total RNA of liver tissue and LX-2 cells were separately extracted by Trizol reagent, quantified by spectrophotometry, reverse-transcribed to cDNA by reverse transcription system, and amplified by PCR mix with specific oligonucleotide primers for target sequences and β-actin, which served as internal control. The primers of hepatic markers were: eNOS forward 5'-TGACCCTCACCGATACAACA-3', reverse 5'-CTGGCCTTCTGCTCATTTTC-3'; iNOS forward 5’-GCCTCCCTCTGGAAAGA-3’, reverse 5’-TCCATGCAGACAACCTT-3’; Collagen I forward 5'-AGGCATAAAGGGTCATCGTG-3', reverse 5'-ACCGTTGAGTCCATCTTTGC-3'; β-actin forward 5’-AGCCATGTACGTAGCCATCC-3’, reverse 5’-CTCTCAGCTGTGGTGGTGAA -3’. And the primers of cellular markers were: eNOS forward 5'-CCCTCACCGCTACAACATC-3', reverse 5'-GCTCATTCTCCAGGTGCTTC-3'; iNOS forward 5’-GTGGAAGCGGTAACAAAGGA-3’, reverse 5’-TGCCATTGTTGGTGGAGTAA -3’; α-SMA forward 5’-TTCAATGTCCCAGCCATGTA-3’, reverse 5’-GAAGGAA TAGCCACGCTCAG-3’; Collagen I forward 5'-CCAAATCTGTCTCCCCAGAA-3', reverse 5'-TCAAAAACGAAGGGGAGATG-3'; β-actin forward 5’-GGACTTCGA GCAAGAGATGG-3’, reverse 5’-AGCACTGTGTTGGCGTACAG-3’. Threshold cycles (Ct) values of target genes were calculated and normalized to that of β-actin.

### Western blot analysis

Total protein of liver tissue and LX-2 cells were extracted and quantified respectively. After sodium dodecyl sulfate-PAGE, the protein was transferred from gel to Polyvinylidene fluoride membrane by semi-dry transfer method. The membrane was blocked and incubated with antibodies against eNOS, iNOS, α-SMA, Collagen I, and β-actin overnight at 4°C. Then membrane was incubated with secondary antibodies and the protein was detected with enhanced chemiluminescence kit. Bio-Profil system was used to analyze the relative quantization of target protein.

### Statistical analysis

The results were expressed as the mean ± standard deviation and were analyzed using one-way ANOVA or Student-Newman-Keuls test. A *P* value of less than 0.05 was considered as statistically significant.

## Results

### Simvastatin decreased the levels of serum ALT, AST, TC, and TG

The levels of serum ALT, AST, TC, and TG were all increased with the consumption of high-fat diet. Compared with rats in the 24^th^ week group, the levels of ALT, AST, TC, and TG were all declined in rats of simvastatin-treatment group (*P*<0.05) (Table 1).

**Table 1:** Serum biological examinations in each group of rats.

Group	n	ALT (U/L)	AST (U/L)	TC (mmol/L)	TG (mmol/L)
Control	12	45.30±11.35	71.80±9.46	1.81±0.20	0.41±0.10
8^th^ week	6	65.00±10.39^#^*	93.17±14.27^#^*	2.77±0.31^#^*	0.69±0.12^#^*
12^th^ week	6	105.50±13.74^#^*	128.67±14.92^#^*	3.45±0.32^#^*	0.86±0.10^#^*
16^th^ week	6	120.50±11.06^#^*	149.50±9.20^#^*	3.85±0.19^#^*	1.02±0.13^#^*
20^th^ week	6	138.67±9.63^#^*	178.00±8.07^#^*	4.15±0.23^#^*	1.24±0.18^#^*
24^th^ week	6	163.00±17.34^#^	193.00±8.85^#^	4.51±0.21^#^	1.41±0.08^#^
Simvastatin	6	132.67±7.09^#^*	152.50±13.32^#^*	4.20±0.23^#^*	1.25±0.10^#^*
*F* value		92.92	110.89	62.70	36.27
*P* value		<0.01	<0.01	<0.01	<0.01

^#^ *P*<0.05, compared with the control group; *P<0.05, compared with 24^th^ week group

### Simvastatin improved the hepatic pathological changes

**Figure 1:**
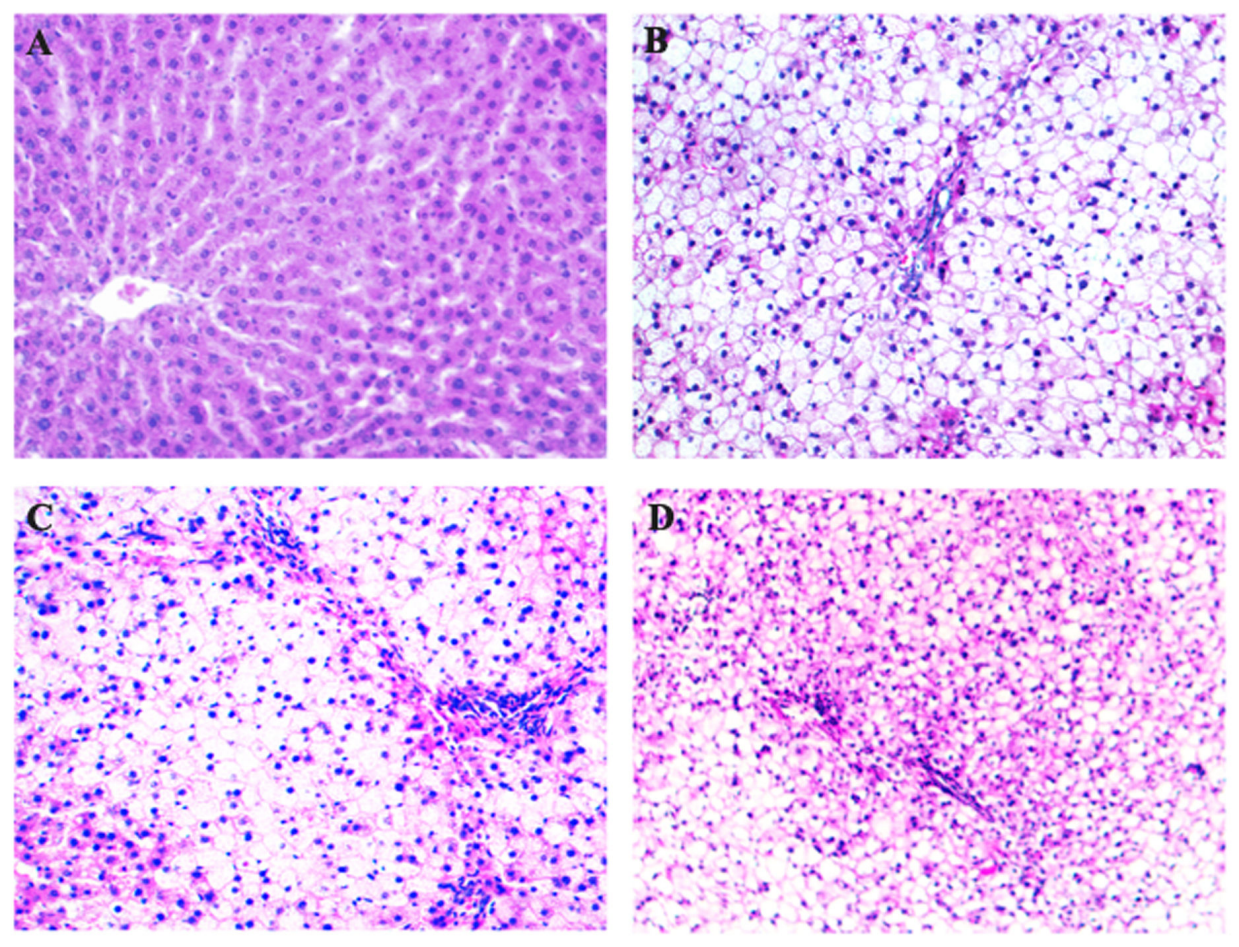
Hematoxylin-eosin staining of hepatic tissues in each group of rats. With the consumption of high-fat diet, hepatocellular steatosis, ballooning degeneration, lobular inflammation, spotty focal necrosis were gradually shown in the hepatic lobule, especially in zone 3 of acinus. At the end of 8^th^ week, simple steatosis appeared with no inflammatory cell infiltration. At the end of 16^th^ week, steatohepatitis established with inflammatory cell infiltration and spotty focal necrosis (B). At the end of 24^th^ week, numerous polymorphs and mononuclear cells infiltration and portal inflammation were frequently observed (C). These changes were improved in the liver of simvastatin-treated rats (D). A: Control ×200; B: 16^th^ week ×200; C: 24^th^ week ×200; D: Simvastatin-treated group ×200.

**Figure 2:**
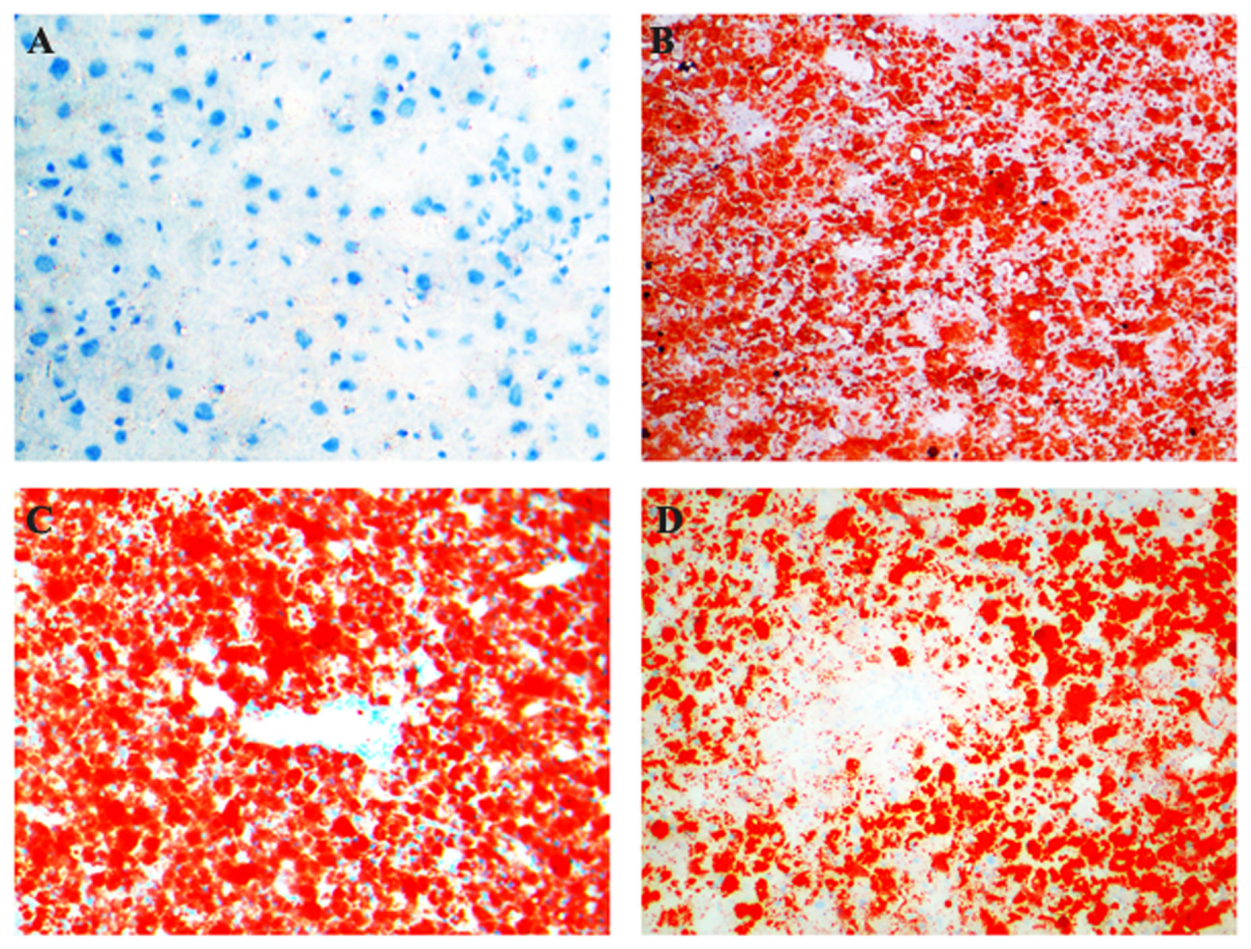
Sudan IV staining of hepatic tissues in each group of rats. Hepatic steatosis emerged until 8^th^ week and aggravated gradually with the consumption of high-fat diet (B). Panacinar steatosis was observed at the end of 24^th^ week (C). These changes were improved in the liver of simvastatin-treated rats (D). A: Control ×200; B: 16^th^ week ×200; C: 24^th^ week ×200; D: Simvastatin-treated group ×200.

**Figure 3:**
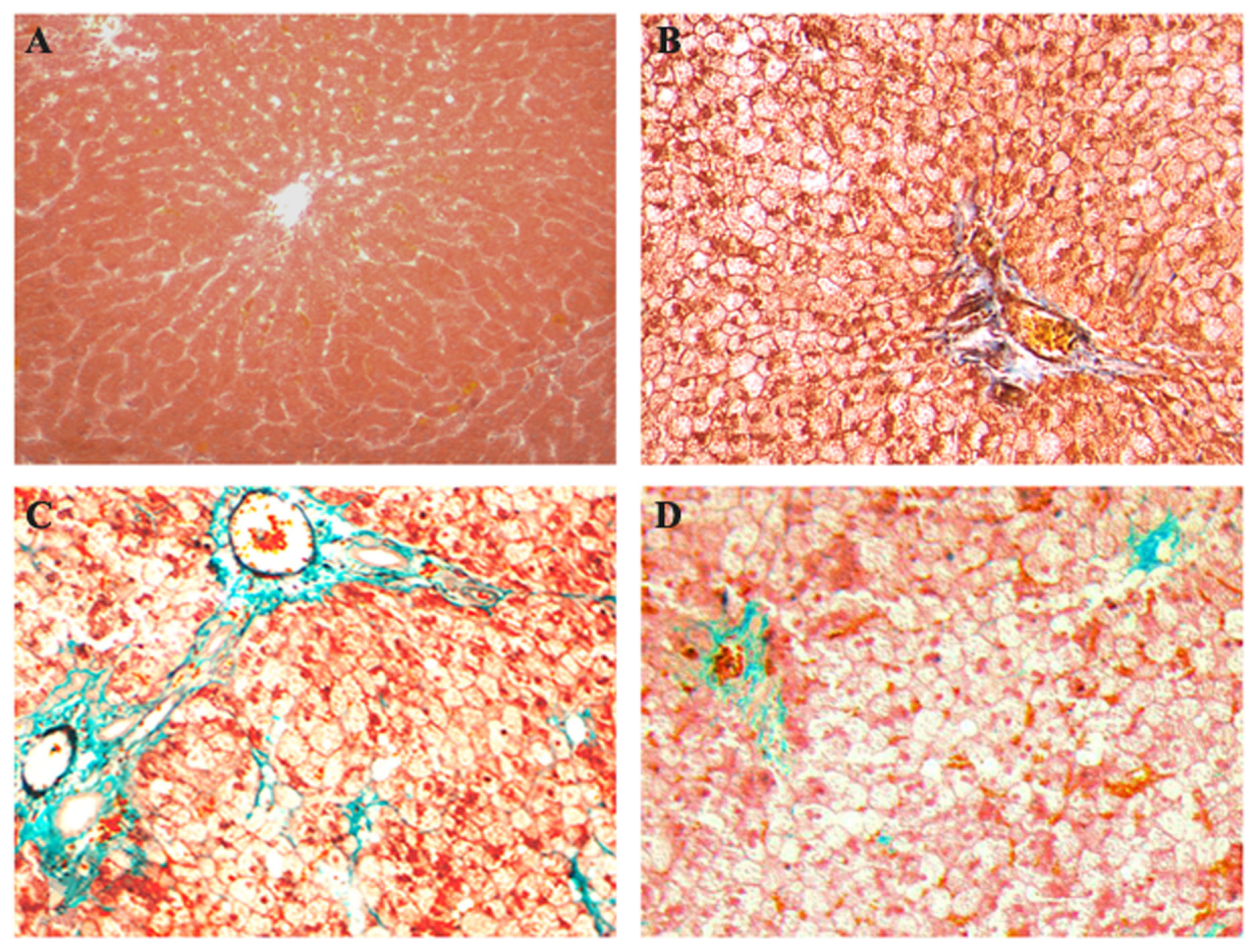
Masson staining of hepatic tissues in each group of rats. Slightly sinusoidal fibrosis appeared only in part of model rats until 16^th^ week (B). At the end of 24^th^ week, all model rats showed hepatic fibrosis in sinusoids, partly in portal area (C). These changes were improved in the liver of simvastatin-treated rats (D). A: Control ×200; B: 16^th^ week ×200; C: 24^th^ week ×200; D: Simvastatin-treated group ×200.

With the consumption of high-fat diet, hepatic steatosis, inflammation, and fibrosis were all aggravated. Hepatic histopathological observation revealed that steatosis appeared at the end of 8^th^ week, steatohepatitis established at the end of 16^th^ week (Figures 1 and 2). Hepatocellular ballooning degeneration, inflammatory cells infiltration, spotty focal necrosis, moderate or severe hepatocellular steatosis were shown in the hepatic lobule, especially in zone 3 of acinus (Figures 1 and 2). At 16^th^ week, part of model rats showed slightly fibrosis in sinusoids. At 24^th^ week, all model rats exhibited sinusoidal fibrosis (Figure 3). These conditions were improved in rats of simvastatin-treatment group (Figures 1D, 2D, and 3D).

### Simvastatin increased the hepatic expression of eNOS and decreased the hepatic expression of iNOS and Collagen I both in mRNA and protein levels

**Figure 4:**
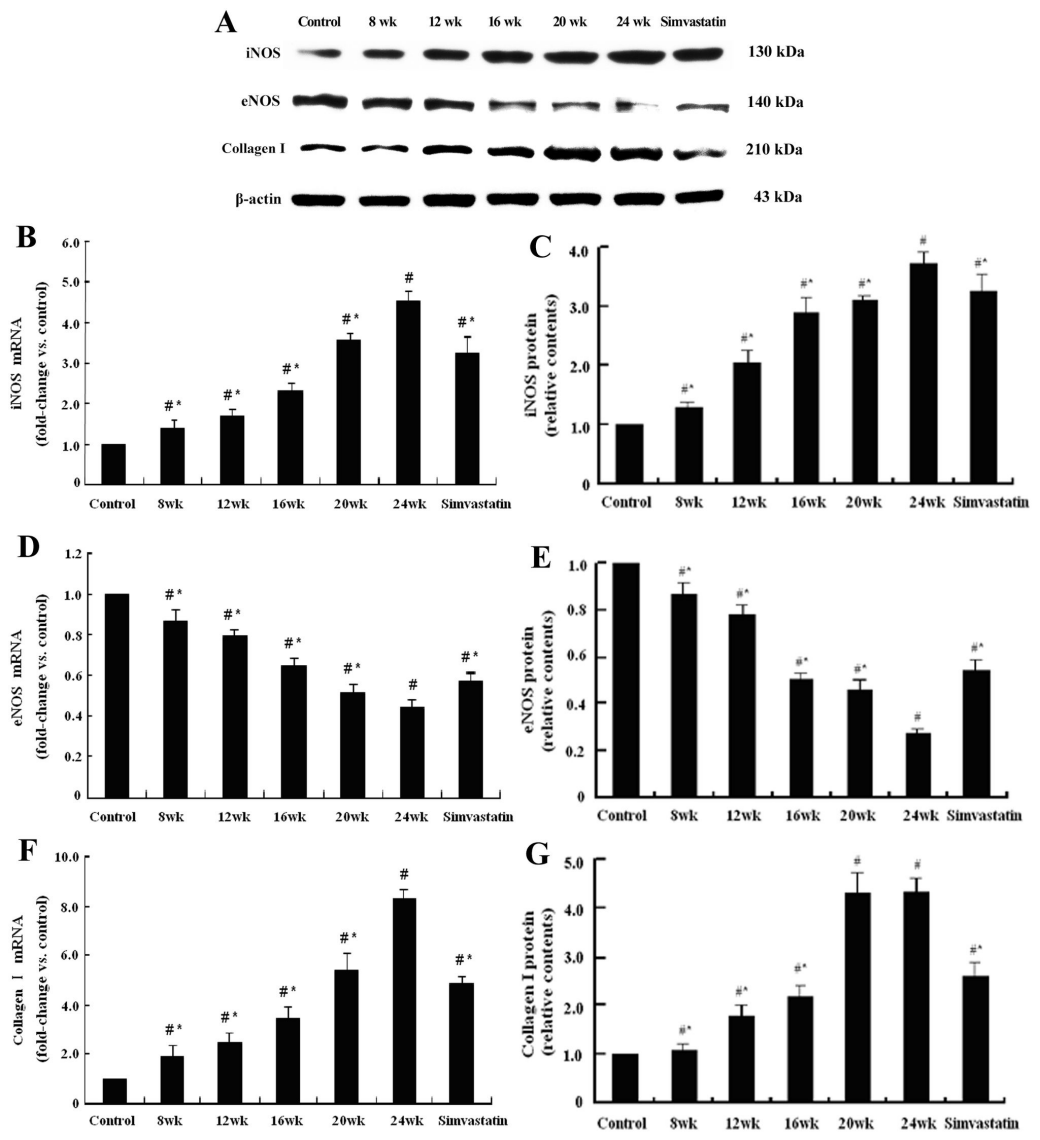
Representative graphs and bar charts of hepatic mRNA and protein expressions of iNOS, eNOS, and Collagen І in each group of rats. The hepatic protein expression of iNOS and eNOS were detected in the control group of rats, with little collagen I expression (A). With the consumption of high-fat diet, the iNOS and collagen I expressions were gradually increased (B, C, F, G), while eNOS expression was gradually decreased (D, E). Compared with rats in the 24^th^ week group, simvastatin treatment could up-regulate eNOS expression (A, D, E) and down-regulate iNOS expression (B, C), followed by improved hepatic fibrosis, which represented by decreased collagen I expression (F, G). (^#^ *P*<0.05, compared with the control group; * *P*<0.05, compared with 24^th^ week group.).

With the progress of NASH, hepatic expression of eNOS both in mRNA and protein levels in model rats were gradually decreased compared with that in the control group (*P*<0.05), while the expression of iNOS and Collagen I were gradually increased (*P*<0.05). Compared with rats only fed with high-fat diet for 24 weeks, rats treated with simvastatin had higher mRNA expression of eNOS (1.30 folds, *P*<0.05), while iNOS (0.72 fold, *P*<0.05) and Collagen I (0.58 fold, *P*<0.05) expressions were lower. The changes of protein expressions were consistent with that of genes (*P*<0.05) (Figure 4).

Simvastatin inhibited the activation of quiescent LX-2 cells, increased the cellular expression of eNOS and decrease the cellular expression of iNOS both in mRNA and protein levels

It had been reported that ADM could convert active HSC into quiescent phenotype [24], which was also confirmed by our former studies [25]. In this study, LX-2 cells approximately acquired quiescent phenotypes by culturing with ADM for three days. These cells were used for investigating the molecular mechanism of HSC activation.

TGF-β1 plays a critical role in the initiation, promotion, and progression of HSC activation and contributes to increased synthesis of myofibroblast phenotype marker gene α-SMA and ECM components such as Collagen І. Therefore, TGF-β1 was selected as a positive control in our study to explore whether simvastatin could inhibit HSC activation. The results showed that α-SMA and Collagen І mRNA and protein expression of quiescent LX-2 cells treated with TGF-β1 were 3 folds higher than that of the control (all *P*<0.05). And compared with the TGF-β1 group, simvastatin could reduce the expression of α-SMA and Collagen І (all *P*<0.05).

**Figure 5:**
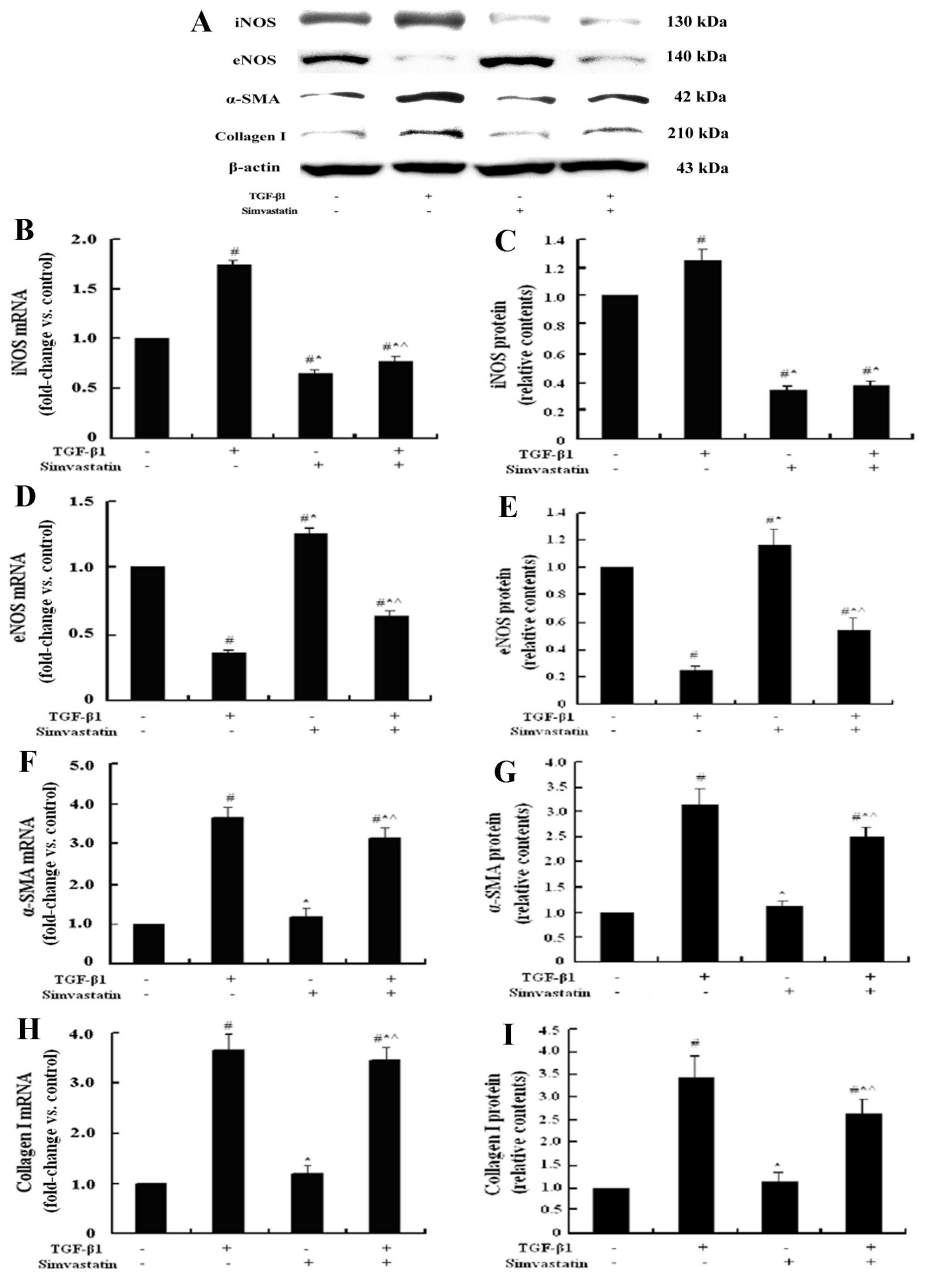
Representative graphs and bar charts of the mRNA and protein expressions of iNOS, eNOS, α-SMA, and Collagen І in LX-2 cells treated with TGF-β1 and simvastatin. Most LX-2 cells obtained the quiescent phenotype after treated with ADM for three days. In LX-2 cells pre-treated with ADM, the mRNA and protein expressions of iNOS and eNOS were detected, with little α-SMA, and Collagen І expressions (A). TGF-β1 behaved as the most powerful activator of HSC and increased the iNOS expression and decreased the eNOS expression (B~E). Compared with the TGF-β1 group of LX-2 cells, LX-2 cells cultured with simvastatin alone or with both simvastatin and TGF-β1 had less iNOS, α-SMA, and Collagen І expressions (B, C, F~I) and more eNOS expression (D, E). (^#^ *P*<0.05, compared with the control group; * *P*<0.05, compared with LX-2 cells treated with TGF-β1; ^ *P*<0.05, compared with LX-2 cells treated with simvastatin.).

Compared with the control group, simvastatin-treated quiescent LX-2 cells had higher eNOS mRNA and protein expressions (1.29 folds and 1.16 folds, respectively, *P*<0.05), lower iNOS expression (0.61 folds and 0.35 folds, respectively, *P*<0.05), and similar α-SMA and Collagen І expressions, with no significant differences (Figure 5).

Furthermore, simvastatin inhibited the activation of LX-2 cells induced by TGF-β1. Compared with the TGF-β1 group, simvastatin plus TGF-β1 group expressed less α-SMA (0.79 folds and 0.86 folds, respectively, *P*<0.05), Collagen І (0.79 folds and 0.82 folds, respectively, *P*<0.05), and iNOS (0.30 folds and 0.45 folds, respectively, *P*<0.05), and more eNOS (2.18 folds and 1.73 folds, respectively, *P*<0.05), both in mRNA and protein levels.

### Simvastatin antagonized the decreased expression of eNOS mediated by L-NAME

L-NAME serves as a competitive inhibitor of NOS, especially selective inhibitor of the constitutive isoforms, which mainly refer to eNOS in the liver tissue. In our study, L-NAME was used to inhibit the expression of eNOS and to demonstrate the important role of eNOS in the mechanism of NASH-related fibrosis both in vivo and in vitro. Compared with the control cells, L-NAME treated quiescent LX-2 cells had higher mRNA and protein expressions of α-SMA (3.46 folds; 3.00 folds, *P*<0.05) and Collagen І (3.53 folds; 3.10 folds, *P*<0.05), and lower eNOS (0.27 fold; 0.27 fold, *P*<0.05) and iNOS expressions (0.60 fold; 0.40 fold, *P*<0.05), which were mostly similar to that of TGF-β1 group (a positive control), except iNOS expression.

**Figure 6:**
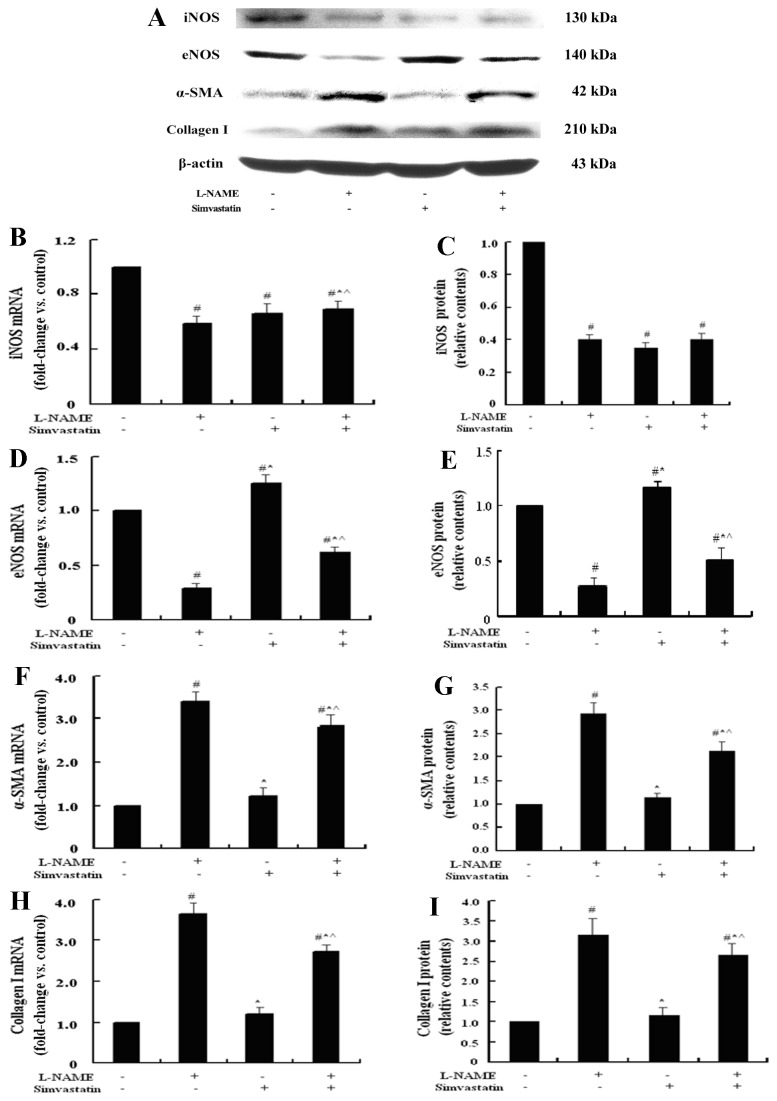
Representative graphs and bar charts of the mRNA and protein expressions of iNOS, eNOS, α-SMA, and Collagen І in LX-2 cells cultured with L-NAME and simvastatin. L-NAME served as a NOS inhibitor, it inhibited both iNOS and eNOS expression (A~E) and induced more α-SMA, and Collagen І expressions (F~I). Simvastatin had antagonistic effect on the activation of LX-2 cells induced by L-NAME via increasing the eNOS expression (D, E). (^#^ *P*<0.05, compared with the control group; * *P*<0.05, compared with LX-2 cells treated with L-NAME; ^ *P*<0.05, compared with LX-2 cells treated with simvastatin.).

Simvastatin could reverse these changes caused by L-NAME in both mRNA and protein levels. Compared with the L-NAME group, simvastatin plus L-NAME could decrease the expression of α-SMA (0.75 fold and 0.72 fold, respectively, *P*<0.05) and Collagen І (0.77 fold and 0.84 fold, respectively, *P*<0.05), increase the expression of eNOS (2.44 folds and 1.92 folds, respectively, *P*<0.05) (Figure 6).

## Discussion

NAFLD is a complex disorder and is recognized as the hepatic manifestation of metabolic syndrome and insulin resistance [26,27]. It is considered as a progressive liver disease which has the potential to progress to cirrhosis and hepatocellular carcinoma (HCC). Recent studies demonstrated that NASH may be a leading cause of cryptogenic cirrhosis, so effective therapies are needed to ameliorate hepatic steatosis, inflammation, and fibrosis, and to prevent the progression to cirrhosis and HCC. In addition, patients with NAFLD and NASH are at increased risk for cardiovascular disease and dyslipidemia treatments should be offered to reduce the cardiovascular risk. Therefore, statins are thought to be available to the management of NAFLD [28].

Simvastatin is one of 3-hydroxy-3-methylglutaryl coenzyme A (HMG-CoA) reductase inhibitors which have lipid-lowering property. In addition, it also exhibits potential benefits such as anti-inflammation, anti-oxidation, improving endothelial dysfunction, increasing nitric oxide bioavailability, stabilizing the atherosclerotic-plaques, decreasing intrahepatic vascular resistance, and reducing portal pressure [29,30]. Therefore, simvastatin may be an optional treatment in patients with NAFLD [31]. Recent studies mostly demonstrated that simvastatin not only reduced circulating low-density lipoprotein cholesterol but decreased hepatic lipid deposition in patients with NASH. However, it was still controversial whether it improved hepatic inflammation and fibrosis [5,19,32]. Nelson A et al. considered that monotherapy with simvastatin does not seem to be an effective treatment for NASH [5]. In their study, a total of 16 patients were enrolled and only 10 patients received simvastatin therapy. If this study enrolled more patients, the result would be different. Additionally, the objects of this study are different from ours, with patients in the former and rats models in the later. The results may be influenced by different study subjects. Zamin Jr I et al. observed that simvastatin could elevate the levels of ALT and AST [32]. In their study, Wistar rats, the same as ours, were used to establish the NAFLD models. But the diets (MCD diet vs. high-fat diet), the dose of simvastatin (0.5 mg/kg/day vs. 4 mg/kg/day), and the course of treatment (started from beginning with the duration of 90 days vs. started from 16^th^ weeks with the duration of 8 weeks) were all different with our study. Therefore, the results of these two studies could not be compared. In this study, simvastatin treatment could ameliorate the progressive hepatic steatosis, inflammation, and fibrosis induced by high-fat diet, indicating that simvastatin had protective effects on the pathogenesis of NASH. Furthermore, cultured quiescent HSC were used to demonstrate whether simvastatin directly improved the hepatic fibrosis besides the indirect function of reduced lipid deposition. The results showed that simvastatin inhibited the activation of HSC and kept the low cellular expressions of α-SMA and Collagen І, suggesting that simvastatin could directly contribute to improvement of hepatic fibrosis.

Since we proved that simvastatin had anti-fibrosis property, the specific mechanism of fibrotic inhibition is further needed to better understand its efficacy. Some studies observed that silent information regulator 1 (SIRT1) and SIRT1-related microRNA-34a were expressed in endothelial progenitor cells obtained from patients with coronary artery disease, and microRNA-34a level was higher than that in non-coronary artery disease subjects. Atorvastatin could contribute to the beneficial effects on endothelial function by up-regulating SIRT1 expression via inhibiting microRNA-34a expression [33]. Another study demonstrated that SIRT1/FoxO3a binding was enhanced in rheumatoid arthritis synovial fibroblasts and simvastatin could increase SIRT1 expression, indicating that simvastatin might be beneficial to inflammatory arthritis through its up-regulation of SIRT1/FoxO3a signaling in synovial fibroblasts [34]. In addition, with the progress of NAFLD, microRNA-34a, apoptosis and acetylated p53 were found to be increased gradually in the hepatic tissues, while SIRT1 decreased [35]. Therefore, statins, particularly simvastatin, might play a crucial role in ameliorating the progression of NAFLD, such as hepatic steatosis, inflammation and fibrosis, via regulating microRNA-34a/Sirtuin-1 pathway.

Recent studies have reported that simvastatin decreased hepatic venous pressure gradient and improved liver perfusion in patients with cirrhosis [36]. And simvastatin attenuated pressure response to volume expansion and improved the vascular disturbances contribute to portal hypertension by selectively increased eNOS expression and NO availability in cirrhotic liver [37]. In vitro, simvastatin inhibited the proliferation of HSC and decrease the expressions of collagen I, III, and IV, but the specific mechanism was unclear [20]. So whether this effect is associated with the regulation of NOS needs to elucidate. iNOS can be increased by many inflammatory agents. In our study, TGF-β1 could activate the quiescent LX-2 cells by increasing the expression of iNOS and decreasing the expression of eNOS. L-NAME, with a dose of 100 µM, was used to inhibit the expression of both eNOS and iNOS, especially eNOS. And we observed that L-NAME activated HSC by decreasing the expression of eNOS and iNOS. Therefore, eNOS was considered to play a more important role than iNOS in the mechanism of HSC’s activation. We found that simvastatin increased eNOS expression and decreased iNOS expression both in hepatic tissue and in cultured LX-2 cells. And simvastatin could attenuat L-NAME’s effect on eNOS, increased eNOS expression, and maintained the low level expression of iNOS. Therefore, simvastatin inhibited the activation of quiescent LX-2 cells by regulating the NOS expression.

## Conclusions

Simvastatin ameliorates the progression of NASH-related hepatic fibrosis. It decreases the hepatic lipid deposition, attenuates hepatic inflammation, and inhibits the development of fibrosis by inhibiting the activation of HSC via modulating iNOS and eNOS. The beneficial effects of simvastatin should be further confirmed in long-term clinical trials for NASH-related fibrosis.
